# Cancer and Risk of COVID‐19 Through a General Community Survey

**DOI:** 10.1634/theoncologist.2020-0572

**Published:** 2020-09-07

**Authors:** Karla A. Lee, Wenjie Ma, Daniel R. Sikavi, David A. Drew, Long H. Nguyen, Ruth C. E. Bowyer, M. Jorge Cardoso, Tove Fall, Maxim B. Freidin, Maria Gomez, Mark Graham, Chuan‐Guo Guo, Amit D. Joshi, Sohee Kwon, Chun‐Han Lo, Mary Ni Lochlainn, Cristina Menni, Benjamin Murray, Raaj Mehta, Mingyang Song, Carole H. Sudre, Veronique Bataille, Thomas Varsavsky, Alessia Visconti, Paul W. Franks, Jonathan Wolf, Claire J. Steves, Sebastien Ourselin, Tim D. Spector, Andrew T. Chan, the COPE consortium

**Affiliations:** ^1^ Department of Twin Research and Genetic Epidemiology, King's College London London United Kingdom; ^2^ School of Biomedical Engineering & Imaging Sciences, King's College London London United Kingdom; ^3^ Clinical and Translational Epidemiology Unit and Division of Gastroenterology, Massachusetts General Hospital and Harvard Medical School Boston Massachusetts USA; ^4^ Department of Medicine, Massachusetts General Hospital and Harvard Medical School Boston Massachusetts USA; ^5^ Department of Clinical Sciences, Lund University Malmö Sweden; ^6^ Department of Medical Sciences, Molecular Epidemiology and Science for Life Laboratory, Uppsala University Sweden; ^7^ Zoe Global Limited London United Kingdom; ^8^ Department of Immunology and Infectious Disease, Harvard T.H. Chan School of Public Health. Boston Massachusetts USA; ^9^ Broad Institute of MIT and Harvard Cambridge Massachusetts USA

## Abstract

Individuals with cancer may be at high risk for coronavirus disease 2019 (COVID‐19) and adverse outcomes. However, evidence from large population‐based studies examining whether cancer and cancer‐related therapy exacerbates the risk of COVID‐19 infection is still limited. Data were collected from the COVID Symptom Study smartphone application since March 29 through May 8, 2020. Among 23,266 participants with cancer and 1,784,293 without cancer, we documented 10,404 reports of a positive COVID‐19 test. Compared with participants without cancer, those living with cancer had a 60% increased risk of a positive COVID‐19 test. Among patients with cancer, current treatment with chemotherapy or immunotherapy was associated with a 2.2‐fold increased risk of a positive test. The association between cancer and COVID‐19 infection was stronger among participants >65 years and males. Future studies are needed to identify subgroups by tumor types and treatment regimens who are particularly at risk for COVID‐19 infection and adverse outcomes.

## Abstract 2

Patients with cancer may be at a higher risk for COVID‐19; however, supporting evidence from large population‐based studies is still limited. This study examined whether incidence of infection is higher in individuals with cancer.

## Introduction

Individuals with cancer may be at higher risk for coronavirus disease 2019 (COVID‐19). However, much of the available data are limited to small studies conducted among hospitalized patients. Through a large community‐based survey, we sought to determine whether incidence of infection, including milder disease with more limited symptoms, is higher in individuals with cancer, including those on chemotherapy/immunotherapy.

## Methods

We recruited individuals from the general population in the U.K., U.S., and Sweden using The COVID Symptom Study, a freely available smartphone application developed by Zoe Global Ltd. with scientific input from researchers and clinicians at Massachusetts General Hospital and King's College London. The application offers a guided interface to report a range of baseline demographic information and comorbidities, as previously reported [1]. Participants are encouraged to use the application daily to report symptoms and COVID‐19 testing results. We queried if individuals were living with cancer (yes/no) and if they were on chemotherapy or immunotherapy (yes/no) beginning on March 29, 2020. We used multivariable logistic regression models to examine the association between cancer and the risk of a positive COVID‐19 test, adjusting for age, date, country, and additional covariates including sex, body mass index (<18.5, 18.5–24.9, 25–29.9, and ≥30 kg/m^2^), history of diabetes, heart disease, lung disease, kidney disease, and current smoking status (each yes/no). We separately analyzed the risk associated with chemotherapy or immunotherapy for a positive COVID‐19 test among individuals with cancer. Two‐sided *p* values <.05 were considered statistically significant. All analyses were performed using R 3.6.1 (Vienna, Austria).

## Results

Through May 8, 2020, 1,807,559 participants provided demographic and longitudinal symptom and testing information. Compared with individuals without cancer, those with cancer were older, more frequently male, and more commonly overweight or obese, among other comorbidities (Table 1). They were more likely to use several common medications and have health problems requiring them to stay at home. Among 23,266 individuals with cancer and 1,784,293 without cancer, we documented 155 and 10,249 reports of a positive COVID‐19 test, respectively (Table 2). Compared with individuals without cancer, those with cancer had a 60% increased risk of a positive COVID‐19 test (adjusted odds ratio [aOR]: 1.60; 95% confidence interval (CI): 1.36–1.88). The association between cancer and a positive COVID‐19 test was stronger among participants older than 65 years (aOR: 1.93; 95% CI: 1.51–2.46) compared with younger participants (aOR: 1.32; 95% CI: 1.06–1.64; P_interaction_ < .001) and among males (aOR: 1.71; 95% CI: 1.36–2.15) compared with females (aOR: 1.43; 95% CI: 1.14–1.79; P_interaction_ = .02). The risk estimates did not significantly differ according to race (white: aOR: 1.84; 95% CI: 1.52–2.23; nonwhite: aOR: 2.08; 95% CI: 1.05–4.12; P_interaction_ = .85). Additional adjustment for education and income as surrogates for socioeconomic status did not materially change the associations. Chemotherapy or immunotherapy was associated with a twofold increased risk of a positive COVID‐19 test (aOR: 2.22; 95% CI: 1.68–2.94). An increased risk of hospitalization due to COVID‐19 was associated with a cancer diagnosis (aOR: 2.47; 95% CI: 2.22–2.76) and chemotherapy/immunotherapy (aOR: 4.16; 95% CI: 2.50–4.95). Using a validated symptom‐based prediction model for COVID‐19 [2], the aOR for predicted COVID‐19 was 1.32 (95% CI: 1.22–1.42) for those with cancer and 1.55 (95% CI: 1.33–1.79) for those on chemotherapy/immunotherapy. The symptoms were somewhat less prominent in patients with cancer (data not shown).

**Table 1:** Baseline characteristics of participants according to cancer history and chemotherapy or immunotherapy

Characteristics	Cancer, %		Chemotherapy/immunotherapy, %	
	No (*n* = 1,784,293)	Yes (*n* = 23,266)	No (*n* = 1,802,655)	Yes (*n* = 4,904)
Country				
U.K.	81.6	77.1	81.5	75.1
U.S.	11.8	18.3	11.9	19.7
Sweden	6.6	4.7	6.6	5.2
Age group, years				
<25	15.5	0.9	15.3	1.8
25–34	14.1	1.0	14.0	1.7
35–44	17.0	3.9	16.9	5.9
45–54	18.9	10.7	18.8	14.8
55–64	17.3	23.1	17.4	23.4
≥65	17.2	60.3	17.6	52.5
Male sex	42.7	55.2	42.9	45.8
Ethnicity				
Hispanic	5.9	3.5	5.9	4.4
Non‐Hispanic	90.2	93.3	90.2	91.7
Prefer not to say	3.9	3.3	3.9	3.9
Race				
White	93.6	95.6	93.7	94.9
Black	1.4	1.0	1.4	1.1
Asian	2.5	1.7	2.5	2.0
Other	2.0	1.2	2.0	1.4
Prefer not to say	0.4	0.4	0.4	0.5
Body mass index group				
<18.5	6.5	3.3	6.4	4.1
18.5–24.9	40.4	37.0	40.3	38.7
25–29.9	31.1	36.5	31.2	33.8
≥30	22.0	23.2	22.0	23.3
Comorbidities				
Diabetes	4.0	10.2	4.1	10.3
Heart disease	3.4	12.6	3.5	10.6
Lung disease	12.1	17.1	12.1	18.4
Kidney disease	0.8	4.5	0.9	4.8
Smoking status				
Never	70.8	61.6	70.7	63.7
Past	20.2	33.2	20.4	31.4
Current	9.0	5.3	8.9	5.0
Limited mobility^a^	7.1	40.9	7.4	64.1
Medication use				
Immunosuppressants^b^	3.5	16.3	3.5	43.7
ACE inhibitor	7.3	17.1	7.4	15.4
Aspirin	4.8	16.3	4.9	17.5
NSAIDs	7.4	10.8	7.5	10.8
Interaction with individuals with COVID‐19				
No	87.0	93.2	87.1	94.5
Yes, suspected	9.5	4.8	9.4	3.8
Yes, documented	3.5	2.0	3.5	1.7
Frontline health care worker	7.2	2.8	7.1	2.1

Proportions are calculated based on the total number of participants with available data.

History of cancer, uses of aspirin and NSAIDs, and smoking status have been queried since launch in the U.S. and Sweden and since March 29, 2020, in the U.K.

^a^ Immunosuppressant medications including steroids, methotrexate, biologics were asked.

^b^ Limited mobility was asked as “In general, do you have any health problems that require you to stay at home?”

Abbreviations: ACE, angiotensin‐converting enzyme; COVID‐19, coronavirus disease 2019; NSAIDs, nonsteroidal anti‐inflammatory drugs.

**Table 2:** Associations between cancer history, chemotherapy/immunotherapy, and risk of COVID‐19

COVID‐19/cancer status	Event/participants	Odds ratio (95% CI)	
		Model 1	Model 2
Positive COVID‐19 testing			
Living with cancer			
No	10,249/1,784,293	1	1
Yes	155/23,266	1.65 (1.40–1.93)	1.60 (1.36–1.88)
Chemotherapy/immunotherapy			
No	4,854/1,802,655	1	1
Yes	50/4,904	2.34 (1.77–3.09)	2.22 (1.68–2.94)
Predicted COVID‐19 infection			
Living with cancer			
No	83,874/1,784,293	1	1
Yes	725/23,266	1.38 (1.27–1.48)	1.32 (1.22–1.42)
Chemotherapy/immunotherapy			
No	84,403/1,802,655	1	1
Yes	196/4,904	1.61 (1.39–1.86)	1.55 (1.33–1.79)
Hospitalization for COVID‐19			
Living with cancer			
No	11,698/1,784,293	1	1
Yes	370/23,266	2.69 (2.42–2.99)	2.47 (2.22–2.76)
Chemotherapy/immunotherapy			
No	11,928/1,802,655		
Yes	140/4,904	4.62 (3.89–5.49)	4.16 (3.50–4.95)

Model 1: adjusted for age groups, country, and date at entry.

Model 2: further adjusted for body mass index (<18.5, 18.5–24.9, 25–29.9, and ≥ 30 kg/m^2^), sex, history of diabetes, heart disease, lung disease, kidney disease, and current smoker status.

Abbreviations: CI, confidence interval; COVID‐19, coronavirus disease 2019.

## Discussion

Among >1.8 million participants, we found that individuals living with cancer had a 60% increased risk of a positive COVID‐19 test or hospitalization with COVID‐19, with greater risks for older individuals or those receiving anticancer therapies. Prior studies have shown that individuals with cancer make up a disproportionate share of poorer COVID‐19 outcomes [3, 4, 5, 6], including death. However, these studies had small sample sizes and are largely based on hospitalized patients, capturing the most severe cases. Individuals living with cancer also tend to be older with greater comorbidities that predispose to hospitalization and adverse events.

A retrospective cohort study with 1,035 COVID‐19–positive patients with cancer in the U.S., Canada, and Spain reported high 30‐day all‐cause mortality [7]. This study also demonstrated numerically higher rates of death outside the intensive care unit in patients with active cancer, with the reverse pattern seen for those in remission. A prospective cohort study reported that COVID‐19 mortality in 800 U.K.‐based patients with cancer was principally related to advancing age and the presence of other noncancer comorbidities, but not recent anticancer treatment [8]. Our results from a large, community‐based sample support that incidence of infection, including milder disease with more limited symptoms, is also higher in individuals with cancer.

Our study was limited by the use of self‐reported information collected from individuals who used smartphone devices, thereby under‐representing those without smartphones. COVID‐19 testing was not based on uniform screening. However, shortages of polymerase chain reaction–based testing kits in both the U.K. and the U.S. early in the pandemic did not make such an approach feasible. Additionally, we had limited data on specific tumor types and treatment regimen. We are planning future studies collecting more detailed information from individuals with cancer with linkage to other data sources.

## Conclusion

Within a large population‐based sample that encompassed more than 20,000 patients with cancer, we demonstrated a significantly increased risk of COVID‐19 infection among patients with cancer, which was greater among older and male individuals. Treatment with chemotherapy or immunotherapy was associated with increased risk of infection.

## Disclosures

**David A. Drew:** Zoe Global Ltd. (RF); **Jonathan Wolf:** Zoe Global Ltd. (E, OI); **Tim D. Spector:** Zoe Global Ltd. (C/A); **Andrew T. Chan:** Zoe Global Ltd. (RF). The other authors indicated no financial relationships.

(C/A) Consulting/advisory relationship; (RF) Research funding; (E) Employment; (ET) Expert testimony; (H) Honoraria received; (OI) Ownership interests; (IP) Intellectual property rights/inventor/patent holder; (SAB) Scientific advisory board
